# Immune Sensitization to the 60 kD Heat Shock Protein and Pregnancy Outcome

**DOI:** 10.1155/S1064744997000239

**Published:** 1997

**Authors:** A. Neuer, S. Spandorfer, P. Giraldo, C. Mele, H. C. Liu, K. Marzusch, D. Kneissl, S. S. Witkin

**Affiliations:** Division of Immunology and Infectious Diseases Department of Obstetrics and Gynecology Cornell University Medical College 515 East 71st Street New York NY 10021 USA

## Abstract

Heat shock proteins are highly conserved proteins present in organisms ranging from bacteria to man. They are both dominant microbial immunogens and among the first proteins produced during mammalian embryo development. Since bacterial and human heat shock proteins share a high degree of amino acid sequence homology, it has been suggested that sensitization to bacterial heat shock proteins during an infection may result in autoimmunity to human heat shock proteins. Infertile couples seeking in vitro fertilization (IVF) may have been previously sensitized to bacterial heat shock proteins as a consequence of an asymptomatic upper genital tract infection. Due to daily clinical monitoring and precisely timed fertilization these patients are an ideal study group to investigate the effect of prior sensitization to heat shock proteins on preimplantation embryo development and implantation failure. Immune sensitization at the level of the cervix to the 60 kD heat shock protein (hsp60) has been associated with implantation failure in some IVF patients. Similarly, the highest prevalence of circulating hsp60 antibodies among IVF patients was found in the sera of women whose embryos failed to develop in vitro. To more directly assess whether humoral immunity to hsp60 influenced in vitro embryo development, a mouse embryo culture model was established. Monoclonal antibody to mammalian hsp60 markedly impaired mouse embryo development in vitro. These data suggest that immune sensitization to human hsp60, possibly developed as a consequence of infection, may adversely affect pregnancy outcome in some patients.


Infectious Diseases in Obstetrics and Gynecology 5:154–157 (1997)
© 1997 Wiley-Liss, Inc.

### Immune Sensitization to the 60 kD Heat Shock Protein and Pregnancy Outcome

A. Neuer,* S. Spandorfer, P. Giraldo, C. Mele, H.C. Liu, K. Marzusch, D. Kneissl, and S.S. Witkin

*Department of Obstetrics and Gynecology, Cornell University Medical College, New York, NY*

### Abstract

Heat shock proteins are highly conserved proteins present in organisms ranging from bacteria to man. They are both dominant microbial immunogens and among the first proteins produced during mammalian embryo development. Since bacterial and human heat shock proteins share a high degree of amino acid sequence homology, it has been suggested that sensitization to bacterial heat shock proteins during an infection may result in autoimmunity to human heat shock proteins. Infertile couples seeking in vitro fertilization (IVF) may have been previously sensitized to bacterial heat shock proteins as a consequence of an asymptomatic upper genital tract infection. Due to daily clinical monitoring and precisely timed fertilization these patients are an ideal study group to investigate the effect of prior sensitization to heat shock proteins on preimplantation embryo development and implantation failure. Immune sensitization at the level of the cervix to the 60 kD heat shock protein (hsp60) has been associated with implantation failure in some IVF patients. Similarly, the highest prevalence of circulating hsp60 antibodies among IVF patients was found in the sera of women whose embryos failed to develop in vitro. To more directly assess whether humoral immunity to hsp60 influenced in vitro embryo development, a mouse embryo culture model was established. Monoclonal antibody to mammalian hsp60 markedly impaired mouse embryo development in vitro. These data suggest that immune sensitization to human hsp60, possibly developed as a consequence of infection, may adversely affect pregnancy outcome in some patients. Infect. Dis. Obstet. Gynecol. 5:154–157, 1997. © 1997 Wiley-Liss, Inc.

Key words

60 kD heat shock proteins; embryo development; reproductive failure; preimplantation mouse embryo

Under physiological conditions, heat shock proteins (stress response proteins) function as molecular chaperones, mediating the folding and assembly of other intracellular proteins and preventing inappropriate protein aggregation. They also facilitate intracellular protein transport and maintain proteins in an inactive configuration. Under conditions of rapid cell growth or differentiation such as in early pregnancy, or following exposure to an environmental stress such as inflammation, fever, or toxic chemicals, heat shock protein synthesis is markedly increased. Heat shock proteins are classified into different families on the basis of their molecular weight and further distinguished according to their inducibility.

The 60 kD heat shock protein (hsp60) is one of the best characterized molecular chaperones of both eukaryotic and prokaryotic organisms. The

Contract grant sponsor: Deutsche Forschungsgemeinschaft; Contract grant number: Ne 602/1-1; Contract grant sponsor: R.K. Mellon Family Foundation.

*Correspondence to: A. Neuer, Division of Immunology and Infectious Diseases, Department of Obstetrics and Gynecology, Cornell University Medical College, 515 East 71st Street, New York, NY 10021.

Received 1 October 1997

Accepted 21 October 1997

**TABLE 1. Properties of the 60 kD family of heat shock proteins**

| |
|---|
| Detectable in all eukaryotic and prokaryotic organisms. |
| Essential chaperone proteins involved in transport, folding, and assembly of protein subunits. |
| Production is elevated in response to environmental stress factors in order to minimize protein denaturation. |
| Highly conserved amino acid sequence throughout evolution. The human and bacterial proteins share a sequence homology of approximately 50%. |
| Immune responses to conserved regions of heat shock proteins have been implicated in autoimmunity. |

major properties of hsp60 are delineated in Table 1. Studies have revealed that members of the hsp60 family of heat shock proteins are dominant antigens of many pathogenic microorganisms such as *Escherichia coli, Salmonella* spp,[1] and *Chlamydia trachomatis*,[2] the most common pathogen associated with tubal infertility.

### HSP60 AND POSTINFECTIOUS AUTOIMMUNITY: POSSIBLE CONSEQUENCES FOR EARLY PREGNANCY

Bacterial hsp60 is highly immunogenic in man.[3] Typically, during the course of an acute infection immunity is restricted to hsp60 epitopes that are specific to the invading microorganism. However, since bacterial and human heat shock proteins are highly conserved proteins and share approximately a 50% amino acid sequence homology,[4] it has been proposed that a prolonged or repeated bacterial infection can trigger immunity to conserved hsp60 epitopes that are also expressed in man.[1,5] This would result in autoimmunity to human (self) heat shock proteins.

Microbe-induced hsp60 autoimmunity may be inhibitory to the development of early human pregnancy. Many couples with fertility problems, especially women with occluded fallopian tubes, have had a persistent and "silent" *C. trachomatis* genital tract infection.[6,7] Thus, conditions favorable to hsp60 autoimmunity may have been present. Heat shock proteins are also among the first proteins produced during embryogenesis and are essential for embryo development.[8–10] In addition, heat shock proteins are specifically expressed in the human endometrium throughout the menstrual cycle and during the postovulatory implantation phase.[11] Hsp60 expression in the human decidua at 7–11 weeks gestation has been identified,[12,13] thus representing a potential target tissue for cross-reacting antibodies and a source of hsp60 capable of reactivating hsp60-sensitized lymphocytes. A murine hybridoma specific for mammalian hsp60 was shown to react with the surface of murine and human trophoblast, suggesting surface hsp60 expression by these cells as well.[14]

A possible model for impairment of early stage pregnancy after immune sensitization to conserved regions of the *C. trachomatis* hsp60 has been outlined previously.[7,15,16] However, the precise mechanism of hsp60-related immunopathogenesis during pregnancy remains unproven. The direct impairment of fetal development and/or fetal or maternal cell viability by anti-hsp60 antibodies or sensitized lymphocytes, or interference with immune regulatory mechanisms necessary to prevent rejection of the semi-allogenetic embryo, may induce early stage pregnancy loss.

### HSP60 AND IN VITRO FERTILIZATION (IVF) OUTCOME

Women undergoing IVF with evidence of local cervical immunity to the *C. trachomatis* hsp60 had an increased prevalence of unsuccessful outcome compared to antibody negative women.[7] In addition, there was a relation between cervical IgA antibodies to a conserved hsp60 epitope expressed in both the human and chlamydial proteins and the failure of successful implantation after embryo transfer in IVF patients.[15] In other infertility patients who were not undergoing IVF cervical IgA anti-human hsp60 was shown to be associated with a history of recurrent spontaneous abortion.[15] These data implicated a genital tract immune response to conserved regions of hsp60 with early stage pregnancy loss. To further elucidate the possible contribution of anti-hsp60 antibodies to reproductive failure we related the seroprevalence of antibodies to the human hsp60 in growth medium containing maternal serum. The results (Table 2) indicated that serum IgG antibodies to the human hsp60 were significantly ($P = 0.004$) more common in patients with arrested in vitro embryo development than in IVF patients whose embryos continued to grow and were transferred to the uterus.

### MOUSE IN VITRO EMBRYO STUDIES

Most recently, we have investigated the direct effect of antibodies to the mammalian hsp60 on

TABLE 2. Relation between circulating IgG antibodies to hsp60 and IVF outcome[a]

| IVF outcome | No. subjects | No. hsp60+ (%) |
|---|---|---|
| No fertilization | 14 | 1 (7.1) |
| Arrested embryo development | 13 | 6 (46.2)* |
| Embryo transfer | | |
| Not pregnant | 75 | 7 (9.3) |
| Pregnant | 53 | 9 (17.0) |

[a]Sera from 155 women were tested.
*$P = 0.004$ vs. all others.

TABLE 3. In vitro development of mouse embryos in the presence of monoclonal antibodies to mammalian hsp60

| Antibody | No. tested | No. developed/total No. examined (%) | | |
|---|---|---|---|---|
| | | Day 3[a] | Day 5[b] | Day 7[c] |
| None | 112 | 80 (72) | 79 (71) | 79 (71) |
| $IgG_1$ | 62 | 49 (79) | 45 (73) | 41 (66) |
| Hsp60 | 75 | 22 (29)* | 16 (21)* | 21 (28)* |

[a]Blastocyst stage.
[b]Hatched blastocyst stage.
[c]Outgrowth.
*$P < 0.0001$ vs. $IgG_1$.

mouse embryo development in vitro. Six to 8-week-old mice (strain B6D2F1) were superovulated by intraperitoneal injections of pregnant mare serum gonadotropin. After mating, females exhibiting copulation plugs were sacrificed and two-cell embryos were flushed from the oviducts. A total of 249 embryos were transferred to wells of tissue culture plates containing either RPMI 1640 culture medium and 10% fetal calf sera [complete medium, or complete medium plus 100 µg/ml of a monoclonal antibody to mammalian hsp60 (SPA 806, StressGen, Victoria, B.C., Canada)] purified mouse $IgG_1$ (100 µg/ml) as control. Embryo development was evaluated microscopically after 3, 5, and 7 days in culture, and the number of blastocysts, hatched blastocysts, and outgrown trophoblasts was determined.

Inclusion of anti-hsp60 antibody to the culture medium inhibited embryo development at each time period examined. At day 3, only 29% (22/75) of the embryos cultured with this antibody reached the blastocyst stage compared with 72% (80/112) of embryos cultured in medium and 79% (49/62) cultured in medium plus mouse $IgG_1$ ($P < 0.0001$). On day 5, hatched embryos were present in 21% (16/75) of cultures containing anti-hsp60, 71% (79/112) of cultures containing media ($P < 0.0001$), and 73% (45/62) of cultures containing $IgG_1$. At day 7, outgrown trophoblasts were observed in 28% (21/75) of cultures containing anti-hsp60, 71% (79/112) containing media, and 66% (41/62) of cultures with $IgG_1$ ($P < 0.0001$). These results are summarized in Table 3.

### DISCUSSION

During the preimplantation stage of mammalian embryo development many rapid changes occur. After formation of the zygote the genome of the embryo becomes activated and assumes control of subsequent cell division and differentiation. Hsp60 expression has been demonstrated in mouse embryos at this early stage.[9] Heat shock protein gene expression occurs in two-cell embryos concurrent with the onset of zygote gene activation.[8,9] Gene transcription for a hsp70 may be initiated even earlier at the one-cell stage.[17]

Hsp60 is predominately present within mitochondria. However, hsp60 expression at other sites has been consistently observed. Recent immunoelectron microscopic localization studies of hsp60 revealed that in addition to the above-mentioned mitochondrial localization, 15–20% of the total hsp60 was present at discrete extramitochondrial sites including the cell surface.[18] Heat shock proteins are also found on the cell surface of tumor cells where they elicit an anti-tumor immune response.[19]

In our mouse model, anti-hsp60 antibodies exhibited a detrimental effect on in vitro mouse embryo development. The mechanism(s) of anti-hsp60 inhibition of mouse embryo development in vitro is completely unknown. The zona pellucida of mouse oocytes and zygotes is permeable to macromolecules. Molecules up to 170 kD have been shown to penetrate through the zona pellucida of postovulated mouse oocytes.[20] In addition, the permeability of mouse zona pellucida to IgG with the subsequent induction of embryo damage has been demonstrated.[21] The ability of IgG to enter intact cells has also been demonstrated. IgG anti-ribonucleoprotein and IgG anti-DNA were shown to penetrate into epithelial and fibroblast cells where they reacted with intranuclear antigen and induced cell death.[22]

The extent of heat shock protein gene transcription in mouse embryos varies depending upon in vitro culture conditions[23] and the specific inbred strain utilized.[24] Therefore, the direct relevance of the mouse embryo studies to in vitro and in vivo embryo development in man remains to be definitively determined. Since in IVF the in vitro fertilized embryos are most often cultivated in medium containing maternal sera, the effect of serum containing different titers of antibodies to human hsp60 and other heat shock proteins on in vitro embryo growth and in relation to as yet undefined genetic variables should be further investigated. Such studies are now in progress.

In conclusion, these results suggest that a late sequelae of a persistent or chronic genital tract infection may be the development of immune sensitization to conserved epitopes of hsp60. This event might compromise the success of subsequent natural or assisted fertility attempts.

### ACKNOWLEDGMENTS

This study was supported in part by grant Ne 602/1-1 from the Deutsche Forschungsgemeinschaft and by the R.K. Mellon Family Foundation.

### REFERENCES

1. Kaufmann SH, Schoel B, Embden JD, Koga T, Wand-Württenberger A, Munk ME, Steinhoff U: Heat shock protein 60: Implications for pathogenesis of and protection against bacterial infections. Immunol Rev 121:67–90, 1991.
2. Morrison RP, Ballard RJ, Lyng K, Caldwell HD: Chlamydial disease pathogenesis. The 57 kD chlamydial hypersensitivity antigen is a stress response protein. J Exp Med 170:1271–1283, 1989.
3. Kaufmann SH: Heat shock proteins and the immune response. Immunol Today 11:129–136, 1990.
4. Shinnik TM: Heat shock proteins as antigens of bacterial and parasitic pathogens. Curr Top Microbiol Immunol 167:145–160, 1991.
5. Lamb JR, Bal V, Mendez-Samperio P, Mehlert A, So A, Rothbard J, Lindal S, Young RA, Young DB: Stress proteins may provide a link between the immune response to infection and autoimmunity. Intern Immunol 1:191–196, 1989.
6. Cates W, Wasserheit JN: Genital chlamydial infections: Epidemiology and reproductive sequelae. Am J Obstet Gynecol 164:1771–1781, 1991.
7. Witkin SS, Sultan KM, Neal GS, Jeremias J, Grifo JA, Rosenwaks Z: Unsuspected *Chlamydia trachomatis* infections in the female genital tract and in vitro fertilization outcome. Am J Obstet Gynecol 171:1208–1214, 1994.
8. Bensaude O, Babinet C, Morange M, Jacob F: Heat shock proteins, first major products of zygotic gene activity in mouse embryo. Nature 305:331–333, 1983.
9. Bensaude O, Morange M: Spontaneous high expression of heat shock proteins in mouse embryonal cells and ectoderm from day 8 mouse embryo. EMBO J 2:173–177, 1983.
10. Loones MT, Rallu M, Mezger V, Morange M: Hsp gene expression and HSF2 in mouse development. Cell Mol Life Sci 53:179–190, 1997.
11. Tabibzadeh S, Kong QF, Satyaswaroop PG, Babaknia A: Heat shock proteins in human endometrium throughout the menstrual cycle. Hum Reprod 11:633–640, 1996.
12. Mincheva-Nilson L, Baranow V, Yeung MM, Hammarstrom S, Hammarstrom ML: Immunomorphologic studies of human decidua-associated lymphoid cells in normal early pregnancy. J Immunol 152:2020–2032, 1994.
13. Neuer A, Ruck P, Marzusch K, Dietl J, Kaiserling E, Horny HP, Witkin SS: Human heat shock proteins in first trimester human decidua. Infect Dis Obstet Gynecol 4:188–189, 1996.
14. Heyborne K, Fu YX, Nelson A, Farr A, O'Brien R, Born W: Recognition of trophoblasts by $\gamma\delta$ T-cells. J Immunol 153:2918–2926, 1994.
15. Witkin SS, Jeremias J, Neuer A, David S, Kligman I, Toth M, Willner E, Witkin K: Immune recognition of the 60 KD heat shock protein: Implications for subsequent fertility. Infect Dis Obstet Gynecol 4:152–158, 1996.
16. Witkin SS, Neuer A, Jeremias J, Giraldo P, Tolbert V, Kneissl D, Bongiovanni AM: *Chlamydia trachomatis* infection, immunity and pregnancy outcome. Infect Dis Obstet Gynecol xx:xxx–xxx, 1997.
17. Christians E, Michel E, Renard JP: Developmental control of heat shock and chaperone gene expression. Hsp 70 genes and heat shock factors during preimplantation phase of mouse development. Cell Mol Life Sci 53:168–178, 1997.
18. Soltys BJ, Gupta RS: Immunoelectron microscopic localization of the 60-kDa heat shock chaperone protein (hsp60) in mammalian cells. Exp Cell Res 222:16–27, 1996.
19. Multhoff G, Botzler C, Jennen L, Schmidt J, Ellwart J, Issls R: Heat shock protein 72 on tumor cells—A recognition structure for natural killer cells. J Immunol 158:4341–4350, 1997.
20. Legge M: Oocyte and zygote zona pellucida permeability to macromolecules. J Exp Zool 271:145–150, 1995.
21. Sellens MH, Jenkinson EJ: Permeability of the mouse zona pellucida to immunoglobulin. J Reprod Fertil 42:153–157, 1975.
22. Alarcón-Segovia D, Ruiz-Argüelles A, Llorente L: Broken dogma: Penetration of autoantibodies into living cells. Immunol Today 17:163–164, 1996.
23. Christians E, Campion E, Thompson EM, Renard JP: Expression of the hsp70.1 gene, a landmark of early zygotic activity in the mouse embryo, is restricted to the first burst of transcription. Development 121:113–122, 1995.
24. Chastant S, Christians E, Campion E, Renard JP: Quantitative control of gene expression by nucleocytoplasmic interactions in early mouse embryos: Consequences for reprogrammation by nuclear transfer. Mol Reprod Dev 44:423–432, 1996.

## References

[PDF_00359] Alarcon-Segovia D., Ruiz-Argüelles A., Llorente L. (1996). Broken dogma: penetration of autoantibodies into living cells.. Immunol Today.

[PDF_00309] Bensaude O., Babinet C., Morange M., Jacob F. (1983). Heat shock proteins, first major products of zygotic gene activity in mouse embryo.. Nature.

[PDF_00312] Bensaude O., Morange M. (1983). Spontaneous high expression of heat-shock proteins in mouse embryonal carcinoma cells and ectoderm from day 8 mouse embryo.. EMBO J.

[PDF_00302] Cates W., Wasserheit J. N. (1991). Genital chlamydial infections: epidemiology and reproductive sequelae.. Am J Obstet Gynecol.

[PDF_00366] Chastant S., Christians E., Campion E., Renard J. P. (1996). Quantitative control of gene expression by nucleocytoplasmic interactions in early mouse embryos: consequence for reprogrammation by nuclear transfer.. Mol Reprod Dev.

[PDF_00362] Christians E., Campion E., Thompson E. M., Renard J. P. (1995). Expression of the HSP 70.1 gene, a landmark of early zygotic activity in the mouse embryo, is restricted to the first burst of transcription.. Development.

[PDF_00341] Christians E., Michel E., Renard J. P. (1997). Developmental control of heat shock and chaperone gene expression. Hsp 70 genes and heat shock factors during preimplantation phase of mouse development.. Cell Mol Life Sci.

[PDF_00330] Heyborne K., Fu Y. X., Nelson A., Farr A., O'Brien R., Born W. (1994). Recognition of trophoblasts by gamma delta T cells.. J Immunol.

[PDF_00292] Kaufmann S. H. (1990). Heat shock proteins and the immune response.. Immunol Today.

[PDF_00283] Kaufmann S. H., Schoel B., van Embden J. D., Koga T., Wand-Württenberger A., Munk M. E., Steinhoff U. (1991). Heat-shock protein 60: implications for pathogenesis of and protection against bacterial infections.. Immunol Rev.

[PDF_00297] Lamb J. R., Bal V., Mendez-Samperio P., Mehlert A., So A., Rothbard J., Jindal S., Young R. A., Young D. B. (1989). Stress proteins may provide a link between the immune response to infection and autoimmunity.. Int Immunol.

[PDF_00354] Legge M. (1995). Oocyte and zygote zona pellucida permeability to macromolecules.. J Exp Zool.

[PDF_00316] Loones M. T., Rallu M., Mezger V., Morange M. (1997). HSP gene expression and HSF2 in mouse development.. Cell Mol Life Sci.

[PDF_00322] Mincheva-Nilsson L., Baranov V., Yeung M. M., Hammarström S., Hammarström M. L. (1994). Immunomorphologic studies of human decidua-associated lymphoid cells in normal early pregnancy.. J Immunol.

[PDF_00288] Morrison R. P., Belland R. J., Lyng K., Caldwell H. D. (1989). Chlamydial disease pathogenesis. The 57-kD chlamydial hypersensitivity antigen is a stress response protein.. J Exp Med.

[PDF_00350] Multhoff G., Botzler C., Jennen L., Schmidt J., Ellwart J., Issels R. (1997). Heat shock protein 72 on tumor cells: a recognition structure for natural killer cells.. J Immunol.

[PDF_00356] Sellens M. H., Jenkinson E. J. (1975). Permeability of the mouse zona pellucida to immunoglobulin.. J Reprod Fertil.

[PDF_00294] Shinnick T. M. (1991). Heat shock proteins as antigens of bacterial and parasitic pathogens.. Curr Top Microbiol Immunol.

[PDF_00346] Soltys B. J., Gupta R. S. (1996). Immunoelectron microscopic localization of the 60-kDa heat shock chaperonin protein (Hsp60) in mammalian cells.. Exp Cell Res.

[PDF_00319] Tabibzadeh S., Kong Q. F., Satyaswaroop P. G., Babaknia A. (1996). Heat shock proteins in human endometrium throughout the menstrual cycle.. Hum Reprod.

[PDF_00333] Witkin S. S., Jeremias J., Neuer A., David S., Kligman I., Toth M., Willner E., Witkin K. (1996). Immune recognition of the 60kD heat shock protein: implications for subsequent fertility.. Infect Dis Obstet Gynecol.

[PDF_00305] Witkin S. S., Sultan K. M., Neal G. S., Jeremias J., Grifo J. A., Rosenwaks Z. (1994). Unsuspected Chlamydia trachomatis infection and in vitro fertilization outcome.. Am J Obstet Gynecol.

